# Detection of immune-mediated tumour cell death in vivo using Zirconium-89-labeled APOMAB®

**DOI:** 10.1186/s12967-025-06684-z

**Published:** 2025-06-12

**Authors:** Vasilios Liapis, Nicole L. Wittwer, William Tieu, Tessa Gargett, Michael P. Brown, Alexander H. Staudacher

**Affiliations:** 1 Translational Oncology Laboratory, Centre for Cancer Biology, SA Pathology and University of South Australia, Level 9 Bradley Building, North Terrace, Adelaide, SA 5000 Australia; 2 School of Medicine, University of Adelaide, Adelaide, SA 5000 Australia; 3 School of Physical Sciences, University of Adelaide, Adelaide, SA 5005 Australia; 4 Molecular Imaging and Therapy Research Unit (MITRU), South Australian Health and Medical Research Institute (SAHMRI), Adelaide, SA 5000 Australia; 5 Cancer Clinical Trials Unit, Royal Adelaide Hospital, Adelaide, SA 5000 Australia

**Keywords:** ^89^Zirconium, APOMAB, Immunotherapy, CAR-T cell therapy, PET-imaging

## Abstract

**Background:**

Inconsistent responses to anticancer immunotherapies demonstrate the need for non-invasive methods to detect treatment responses earlier than conventional medical imaging methods allow. The chimeric monoclonal antibody, APOMAB®, targets dead tumour cells following DNA-damaging anticancer treatments via binding of the ribonuclear protein, La/SSB, an intracellular protein overexpressed by tumour cells. La/SSB only becomes accessible to APOMAB binding in post-apoptotic necrotic tumour cells.

**Methods:**

We assessed the ability of APOMAB to detect dead tumour cells after immune-mediated cell death. Co-culture of GD2-specific chimeric antigen receptor (CAR) T-cells with GD2-expressing cancer cell lines demonstrated specific and dose-dependent binding of APOMAB to the resulting dead target cells, confirming detection of immune-mediated cell death. Then, using four distinct preclinical tumour models and in a cancer patient, we investigated APOMAB-immunoPET as a technique to detect immune-mediated tumour cell death.

**Results:**

Within days of treatment, APOMAB-immunoPET showed increased tumour uptake of ^89^Zirconium-labelled APOMAB (^89^Zr-APOMAB) after CAR-T cell therapy, immune checkpoint inhibitor (ICI) therapy with and without chemotherapy, and via endogenous T-cell mediated tumour clearance. In a metastatic melanoma patient after ICI therapy, a previously FDG-avid pulmonary tumour reduced in size as tumour ^89^Zr-APOMAB uptake increased over the 12-day scanning period.

**Conclusions:**

This study demonstrates for the first time that not only does radiolabelled APOMAB provide an initial direct measure of the extent of immune-mediated tumour cell death in vivo but also reveals the heterogeneous nature of tumour responses to T-cell based therapies both within and between individuals.

**Supplementary Information:**

The online version contains supplementary material available at 10.1186/s12967-025-06684-z.

## Background

In the last 15 years, immunotherapy has joined surgery, radiotherapy, and chemotherapy as a major modality of anti-cancer treatment. In solid cancer patients, immune checkpoint inhibitor (ICI) therapy has been the most successful kind of immunotherapy and its anti-cancer effects are mediated mainly by CD8^+^ T cells [1]. Despite clinical success manifesting as potential cures in metastatic melanoma patients receiving single agent ipilimumab [2] or combination ICI therapy [3], most patients with other common types of solid cancer such as of the head and neck, lung, oesophagus, stomach, kidney, and bladder, do not benefit to the same extent [4]. Hence, there is considerable interest in developing clinically useful methods for the early detection of tumour response to ICI therapy so that patients who may not benefit are identified while they remain at risk of serious immune-related toxicities [5].

Although tumour PD-L1, tumour mutational burden, microsatellite instability, and mismatch repair deficiency are FDA-approved biomarkers for ICI therapy, and predictive in specific cancer types [6], they may not predict responses in other cancer types. However, relatively small clinical trials of PET-imaging of probes targeting the most studied signalling axis of PD-1/PD-L1, for example, have not yet established widespread clinical utility for this approach [7].

The conventional medical imaging modalities of computed tomography (CT), magnetic resonance imaging (MRI) and ^18^F-FDG positron emission tomography (PET)/CT are used for tumour response evaluation, but up to two or more months may elapse before decisive results are obtained. Moreover, in evaluating tumour responses to ICI therapy, additional uncertainty arises because of the phenomenon of tumour flare or pseudoprogression in which immune-related inflammation produces transient enlargement of tumour lesions. This uncertainty can be resolved by the assessment methods of immune response evaluation criteria in solid tumours (iRECIST) [8] and immune PET response criteria in solid tumours (iPERCIST) [9].

Another potentially curative form of immunotherapy is chimeric antigen receptor (CAR) T-cell therapy, [10] which has resulted in long term survival in patients with refractory and relapsed B-cell malignancies. There is no comparable, approved CAR-T cell therapy for solid cancer patients although CAR-T cell-mediated tumour cell killing remains the major mechanism of action [11]. Like ICI therapy, there is no direct way of measuring early tumour responses with CAR-T cell therapy.

Consequently, there has been increasing interest in the development of selective, robust, and clinically applicable imaging probes of cancer cell death to measure directly anti-cancer treatment responses in vivo [12–14].

Accordingly, we have developed the chimeric monoclonal antibody APOMAB [15], which is derived from the murine monoclonal antibody DAB4 [16] and binds to La/SSB, an intracellular ribonuclear protein essential for life and overexpressed in malignant cells [16]. During apoptosis, La/SSB translocates from the nucleus to cytoplasm where it becomes available for antigen-specific antibody binding only in cancer cells that have lost plasma membrane integrity in the post-apoptotic necrotic state [16–18]. During post-apoptotic necrosis in vitro, it is evident that La/SSB and its bound antibody become ‘fixed’ in the dead cancer cell via a transglutaminase-2 mediated transamidation reaction [17].

In vivo, specific anti-La/SSB antibody binding to tumours is facilitated by the inefficient post-chemotherapy clearance of dead cancer cells in contrast to the rapid and barely detectable clearance of apoptotic cells in normal tissues [19]. The utility of APOMAB as an imaging marker of cancer cell death in vivo depends on the dynamic in vivo process of tumour macrophage-mediated phagocytosis of APOMAB-bound dead cancer cells [19, 20]. The utility of ^89^Zr-labelled APOMAB as an imaging marker of cancer cell death was realized in preclinical immunoPET studies showing that it specifically detected tumour responses to DNA-damaging and cytotoxic chemotherapy [21, 22]. The utility of ^89^Zr-labelled APOMAB has been extended to the clinical domain with the recent completion of a 20-patient phase 1 immunoPET study (Australian and New Zealand Clinical Trials Registry ID ACTRN12620000622909 via www.anzctr.org.au).

Our recent in vitro characterization of APOMAB binding to dead human cancer cells after treatment with cytotoxic drugs and ionizing radiation established that APOMAB co-localized with γ-H2AX, a bona fide marker of double strand DNA breaks (DSB), and that APOMAB binding was most apparent after treatment with DNA-damaging agents, which cause the greatest formation of DSB [23]. In earlier work, we showed that activation of the extrinsic apoptotic pathway via ligation of the Fas receptor with an anti-CD95 monoclonal antibody enabled detection of both activated caspase-3 and γ-H2AX at 5 h after Fas ligation and corresponded to APOMAB binding of the La/SSB antigen [19]. Fas-induced caspase-activated DNase complex [24] results in DSB and contributes to dismantling of apoptotic cells as they lose plasma membrane integrity. Recently, Fas has been identified as critical effector molecule in antigen-specific T-cell mediated killing of tumour cells and in the bystander killing of antigen-negative tumour cells [25].

Therefore, we aimed to investigate whether APOMAB may more broadly detect T-cell induced death of tumour cells. To our knowledge, a radio-imaging marker of immune-mediated tumour cell death has not been characterized. Here, we investigated if radiolabelled APOMAB can detect immune-mediated tumour cell death in vitro and in vivo and if radiolabelled APOMAB uptake by tumours in vivo after immunotherapy including ICI and CAR-T cell therapies was associated with surrounding T-cell infiltrates. After preclinical and clinical ICI therapy, we also evaluated the heterogeneity in tumour uptake of radiolabelled APOMAB..

## Materials and methods

### Cell culture and antibodies

The murine EL4 thymic lymphoma cells, EMT6 mammary carcinoma cells and HEK293T cells were obtained from American Type Culture Collection (ATCC). EL4 cells were cultured in RPMI-1640 (Sigma-Aldrich) containing 5% fetal calf serum (FCS, Bovogen Biologicals). EMT6 cells were cultured in RPMI-1640 with 10% FCS. HEK293T cells were cultured in Dulbecco’s Modified Eagle Medium (DMEM, Sigma), 2 mM Glutamine (Sigma) and 10% FCS. LAN-1 neuroblastoma cells were purchased from Merck and cultured in RPMI and DMEM-F12 media (Gibco), 2 mM Glutamine and 10% FCS. Murine MC-38 colon carcinoma cells were a kind gift from Dr Imran House (Peter Mac Callum Cancer Centre) and cultured in DMEM containing 10% FCS. All cell lines were cultured with streptomycin and penicillin (Sigma-Aldrich, USA). Cells were tested for mycoplasma contamination using the MycoAlert® Mycoplasma Detection Kit (Lonza) and were negative.

The CRISPR/Cas9 EMT6 cell line (EMT6 CRISPR/Cas9) was generated using Sigma’s LV01 control CRISPR plasmid that contains a non-specific guide RNA (gRNA: CGCGATAGCGCGAATATATT). Although the gRNA was designed *not* to allow targeting of the CRISPR/Cas9 system to any sequence in the human, mouse or rat genomes, this construct still allows for expression of Cas9 and GFP. The plasmid was combined with packaging plasmids to generate lentivirus using HEK293T cells. Viral supernatant was added to EMT6 cells with 5 µg/mL polybrene (Sigma). Transduced cells were selected with 1 µg/mL puromycin (Sigma). A single cell sort was performed on selected GFP positive cells using the MoFlo Astrios cell sorter (Beckman Coulter).

The αPD-1 (clone 29F.1A12), αPD-L1 (clone 10F.9G2) antibodies, and isotype control rat IgG2a (clone 2A3) were purchased from Bio X Cell. The production of APOMAB has been described previously [15].

### CAR-T cell production

Blood collection for CAR-T cell manufacture was approved by the Central Adelaide Local Health Network (CALHN) Human Research Ethics Committee (approval number HREC/16/RAH/191). Buffy coats from Australian Red Cross Lifeblood were obtained under an approved waiver of consent. Peripheral blood mononuclear cells (PBMC) were separated from healthy donor buffy coats using density gradient separation. Cells were cultured in Advanced RPMI (ThermoFisher Scientific) with 10% FCS, 10 ng/mL IL-7 and 5 ng/mL IL-15 (Miltenyi) and activated with 1 µg/mL anti-CD3 (OKT3) and anti-CD28 antibodies (15E8) (Miltenyi) for 24 h.

Activated T-cells were transduced with the third-generation GD2-CAR retroviral vector SFG.iCasp9.2A.14g2a.CD28.OX40.CD3zeta (obtained courtesy of Malcolm K. Brenner, Center for Cell and Gene Therapy, Baylor College of Medicine, Houston TX, USA) [26], with viral supernatant harvested from stable PG-13 producer lines using RectroNectin (Takara Bio) coated plates. Cells were expanded for 7 days and cryopreserved. CAR expression was analysed by flow cytometry using a BD Accuri flow cytometer (BD Biosciences) with a 1A7 antibody (anti-idiotypic antibody purified in-house) followed by a phycoerythrin (PE)-conjugated secondary antibody (BD Biosciences). Non-transduced (NT) T-cells were generated as above with omission of the retroviral supernatant.

Human GD2 expression was examined on LAN-1, MC38 and MC38 engineered to express GD2 (MC38-GD2) cells using Alexa Fluor 647 conjugated anti-Human Disialoganglioside GD2 (BD Biosciences) or Alexa Fluor 647 conjugated Mouse IgG2aκ Isotype Control (BD Biosciences).

### Flow cytometry analysis of EL4, LAN-1 and MC38 cells

EL4 cells were treated for 24 h with 0.625 μg/mL of etoposide (Link Healthcare) and cyclophosphamide (Baxter). Cells were stained with 5 μg/mL of αPD-L1 or isotype (as above) for 30 min on ice. Cells were then washed and incubated on ice with 2 μg/mL goat anti-rat IgG2a Alexa Fluor® 488 (ThermoFisher Scientific) for 20 min. Cells were washed and 1 μg/mL of 7-Aminoactinomycin D (7-AAD; ThermoFisher Scientific) was added to detect and exclude dead cells from the analysis by flow cytometry using a BD Accuri flow cytometer. Specific binding was calculated and expressed as a percentage to their isotype control. LAN-1, MC-38 WT and MC38-GD2 cells were co-cultured with NT-T cells or GD2-CAR-T cells at increasing effector:target (E:T) ratios for 48 h. Cells were collected, washed, and incubated with Alexa Fluor 488 conjugated anti-human CD3 and Alexa Fluor 647 conjugated APOMAB (produced in-house) for 30 min. Cells were then washed and labelled with 1 μg/mL of propidium iodide (PI) (ThermoFisher Scientific) and analysed using a BD Accuri flow cytometer.

LAN-1 cells were treated with 5 µg/mL cisplatin (Hospira), 0.5 µg/mL vinorelbine (Hospira), 0.1 µM ABT-737 (MedChemExpress) and 1 µM S63845 (MedChemExpress), or GD2-CAR-T cells at 1:2 E:T ratio for 48 h. Phosphate buffered saline (PBS), dimethyl sulfoxide (DMSO) and NT-T cells were used as controls. The cells were stained for CD3, APOMAB and PI as described above and analysed by flow cytometry.

### Chelator conjugation and radiolabelling of APOMAB with [^89^Zr]Zr^IV^

Chimeric APOMAB was generated by the fusion of the murine DAB4 variable region sequence to the human IgG1 constant region by CSIRO Molecular and Health Technologies (Parkville, VIC, Australia). A stable producer cell line of chDAB4 was generated using the CHO-XL99 expression system at the National Biologics Facility, Australian Institute for Bioengineering and Nanotechnology, University of Queensland (Brisbane, QLD, Australia) for antibody production as described [15]. [^89^Zr]Zr^IV^ oxalate was produced as described previously [21]. Chimeric APOMAB was conjugated to the bifunctional chelator H_3_DFOSqOEt (DFOSq) and radiolabelled with ^89^Zirconium and validated as described previously [27]. A total of 5 MBq/20 µg of ^89^Zr-APOMAB was injected per mouse.

### Animal experiments

#### LAN-1 model

The SAHMRI Animal Ethics Committee (Adelaide, Australia) approved all animal experiments, which were conducted following institutional ethical guidelines (Ethics approval numbers SAM172, SAM-21–112) which comply with the ARRIVE guidelines. Female mice of 6–10 weeks of age were used for experiments. Mice were euthanized when weight loss was greater than 15% compared to day 1 of treatment, or tumor volume was greater than 600 mm^3^.

NOD scid gamma-null (NSG) mice were inoculated subcutaneously in the right flank with 2 × 10^6^ LAN-1 tumour cells in a 1:1 ratio of Matrigel (Falcon) and PBS. After 7 days, mice were injected intravenously with either PBS, 1 × 10^7^ NT-T cells or 1 × 10^7^ GD2-CAR-T cells (day 0), with ^89^Zr-APOMAB injected intravenously the following day (day 1).

#### EMT6 model

Balb/c mice were inoculated subcutaneously in the right flank with 10^6^ parental EMT6 or EMT6 CRISPR/Cas9 cells in PBS. After 7 days mice were injected intravenously with ^89^Zr-APOMAB.

#### EL4 and MC38 models

C57BL/6 J mice were inoculated subcutaneously in the right flank with 10^6^ EL4 or 2 × 10^5^ MC38 tumour cells in PBS. After 7 days, mice bearing EL4 tumours were given intraperitoneal injections of 25 mg/kg cyclophosphamide and 19 mg/kg etoposide (day 0) and either 200 μg of the α-PD-1 antibody or its isotype control on days 0, 3 and 6. ^89^Zr-APOMAB was injected intravenously on day 1. For the MC38 tumour model, 7 days following tumour inoculation (day 0), mice were given 200 μg α-PD-1 or the isotype control antibody by intraperitoneal injection on days 0, 3 and 6. ^89^Zr-APOMAB injected intravenously on day 1.

Mice were monitored daily, and tumour volume measured at least three times per week using electronic callipers with tumour volume determined using the calculation (a^2^ × b)/2, where a is the shortest diameter and b is the longest diameter of the tumour.

### PET scanning and biodistribution

For PET imaging studies, mice were anesthetized with isoflurane and scanned for 5 min using the Albira Si PET-SPECT small animal scanner (Bruker). Regions of interest were manually drawn from the maximum intensity projection (MIP) sections assisted by the automatic 3D using the PMOD imaging suite (PMOD Technologies version 4.2). Tumour margins and thresholds were automatically detected by the software, which included a standardized cut off determined by PMOD and decay correction.

At the end of the experiment, mice were humanely killed by cervical dislocation, the organs removed, weighed and radiation counts were measured using a Hidex gamma-counter (with background and decay correction) to determine the tissue distribution of ^89^Zr-APOMAB antibody.

### Immunofluorescence analysis of tumour tissue

In the EMT6 Cas9 model, mice were administered with 100 µg of APOMAB 24 h prior to euthanasia at defined time points (days 8, 11 and 14 after inoculation). Tumours were removed and snap frozen in Tissue-Tek OCT cryoprotectant (Sakura FineTek). Five micron sections were fixed for 20 min in Cytofix/Cytoperm (BD Biosciences) at room temperature, washed in PBS and blocked using 10% normal goat serum and 1% bovine serum albumin (BSA) in PBS for 1 h. Sections were incubated with 2 µg/mL anti-mouse CD3 (Abcam) or rabbit IgG isotype control overnight at 4 °C. After washing sections were incubated with 4 µg/mL Alexa Fluor 488 conjugated anti-Rabbit IgG (ThermoFisher) to detect CD3 and 4 µg/mL Alexa-Fluor 555 conjugated anti-Human IgG ThermoFisher to detect APOMAB for 1 h at room temperature. Sections were washed, counterstained with 0.5 μg/mL 4′,6-diamidino-2-phenylindole (DAPI) and examined using the Zeiss Axio Scan 7 slide scanner. QuPath 0.4.3 software was used to quantify the percentage of CD3 + T-cells and APOMAB^+^ cells in tumour sections.

### PET/CT imaging of patients

The phase I trial was approved by the Central Adelaide Local Health Network Human Research Ethics Committee and registered as No. 12620000622909 29/05/2020 with the Australian and New Zealand Clinical Trials Registry (ANZCTR) https://www.anzctr.org.au/Trial/Registration/TrialReview.aspx?id=379693.

In this clinical trial, a patient who provided informed consent to participate in the study, was recruited with stage 4 V600E BRAF-mutant melanoma and received standard doses of ipilimumab and nivolumab as ICI therapy commencing 5 days before a 10 mg mass dose injection of 75 MBq of ^89^Zr-APOMAB on day 0. PET/CT imaging were taken over 12 days on a Siemens Biograph mCT Flow after having a standard FDG-PET/CT scan on the same kind of scanner within the previous 50 days. The patient received two subsequent cycles of ipilimumab and nivolumab on days 14 and 35 before symptomatic disease progression became manifest.

### Statistical analysis

Statistical analyses were performed using GraphPad Prism (v8.4). Comparison of groups was performed by two-tailed *t*-test or intergroup comparisons made by two-way Analysis of Variance (ANOVA). Data are shown as mean ± standard error of the mean (SEM). Statistical significance was reached when *p* < 0.05, with * representing *p* < 0.05, ** *p* < 0.01, *** *p* < 0.001 and **** *p* < 0.0001. All methods and studies were performed in accordance with the guidelines and regulations stated by the University of South Australia, the University of Adelaide, SAHMRI and the Royal Adelaide Hospital.

## Results

### CAR-T cell-mediated killing of GD2-expressing cancer cell lines can be detected by APOMAB

**Fig. 1:**
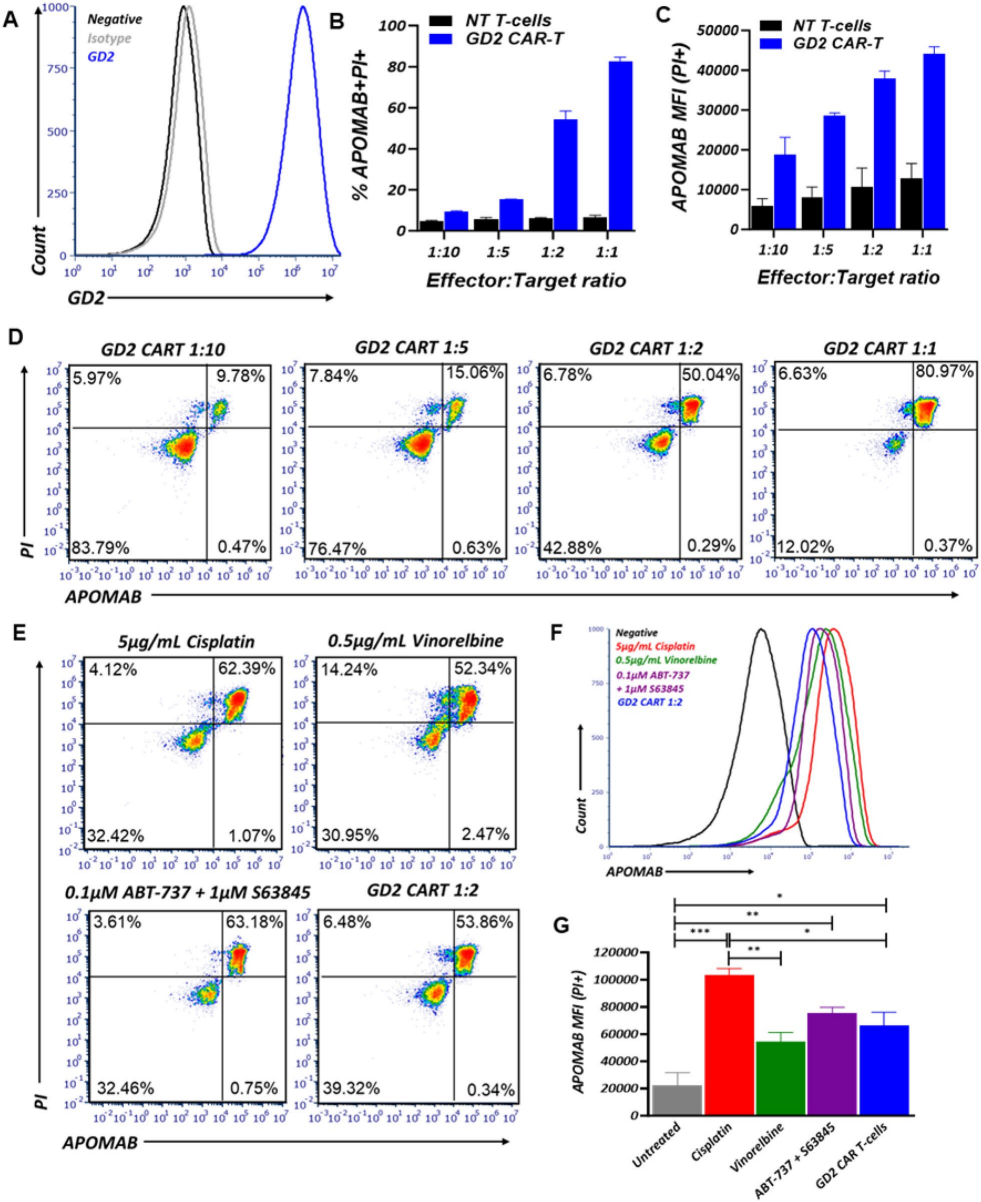
CAR-T cell mediated killing of GD2 expressing cancer cell lines can be detected by APOMAB. APOMAB binding to CAR-T cell mediated cell death was investigated in vitro using the GD2 expressing human neuroblastoma cell line LAN-1. **A** GD2 expression on LAN-1 cells was confirmed using flow cytometry. **B** LAN-1 cells were treated with increasing numbers of NT-T cells or GD2 targeting CAR T-cells for 48 h and analysed by flow cytometry for APOMAB binding. Data presented as percentage of CD3^−^PI ^+^APOMAB^+^ cells, **C** APOMAB median fluorescence intensity (MFI) and **D** representative flow plots. Mean ± SD. One representative experiment of n = 3, with three different donor PBMC. LAN-1 cells were treated with 5 µg/mL cisplatin, 0.5 µg/mL vinorelbine, 0.1 µM ABT-737 + 1 µM S63845 and 1:2 GD2-CAR-T cells for 48 h and analysed by flow cytometry for APOMAB binding. **E** Data are presented as representative flow plots of CD3^−^PI ^+^APOMAB^+^ cells, **F** overlayed histograms of APOMAB binding and **G** APOMAB MFI on PI^+^ cells. Mean ± SEM, n = 3. *p* values were determined by one-way ANOVA * = *p* ≤ 0.05, ** = *p* ≤ 0.01, *** = *p* ≤ 0.001

APOMAB has been shown to bind to cancer cells that have been killed by DNA-damaging anti-cancer therapies [17, 22, 23], but the ability of APOMAB to detect immune-mediated tumour cell death has not yet been established. After confirmation of endogenous GD2 expression on the human neuroblastoma cell line LAN-1 by flow cytometry (Fig. 1A), LAN-1 cells were co-cultured with non-transduced (NT)-T cells or GD2-CAR-T cells at increasing E:T ratios for 48 h and analysed by flow cytometry for APOMAB binding. As the effector to target (E:T) ratio increased, both the percentage of APOMAB^+^ cells (Fig. 1B and D) and the intensity of APOMAB staining increased (Fig. 1C) following co-culture with GD2-CAR T-cells, It is also noted that as E:T ratios increased, non-antigen-specific cancer cell killing by non-transduced T-cells also increased thus resulting in lower level increases in APOMAB MFI. Co-staining with CD3 and PI confirmed APOMAB staining was specific for dead tumour cells only. The specificity of APOMAB binding for GD2-CAR-T cell-induced tumour cell death was confirmed using a second model in which murine MC38 colorectal cancer cells were engineered to ectopically express GD2 (Supplementary Fig. 1A). Co-culture of GD2-CAR T-cells with MC38 WT and MC38 GD2 cells demonstrated induction of tumour cell death and APOMAB binding only when GD2 was expressed (Supplementary Fig. 1B–C).

We noted that the per cell binding of APOMAB (expressed as APOMAB MFI) after CAR-T cell-induced tumour cell death was lower than we had previously observed after cell death induced by DNA-damaging drugs. To better understand the relative avidity of APOMAB binding for dead cancer cells killed by different anti-cancer treatments, we compared four treatment methods based on different mechanisms of action at doses that induce 50–65% cell death after 48 h. LAN-1 cells were treated with the radiomimetic and DNA-crosslinking drug, cisplatin, the tubulin polymerization inhibiting drug, vinorelbine, the combination of BH3 only mimetic drugs, S63845 (MCL-1 inhibitor) and ABT-737 (inhibitor of BCL-2 and BCL-XL), and GD2-CAR-T cells. It is evident that the increased intensity of CAR-T cell killing at higher E:T ratios resulted in greater APOMAB binding (Fig. 1B–D), the level of which was comparable to that elicited by the pro-apoptotic BH3 only mimetic drugs (Fig. 1F and G). Nonetheless, overall level of APOMAB binding was less for immune-mediated tumour cell death than that caused by a potent DNA-damaging drug like cisplatin (Fig. 1F and G).

### Detection of CAR-T cell-mediated cytotoxicity of LAN-1 tumours by APOMAB-immunoPET

**Fig. 2:**
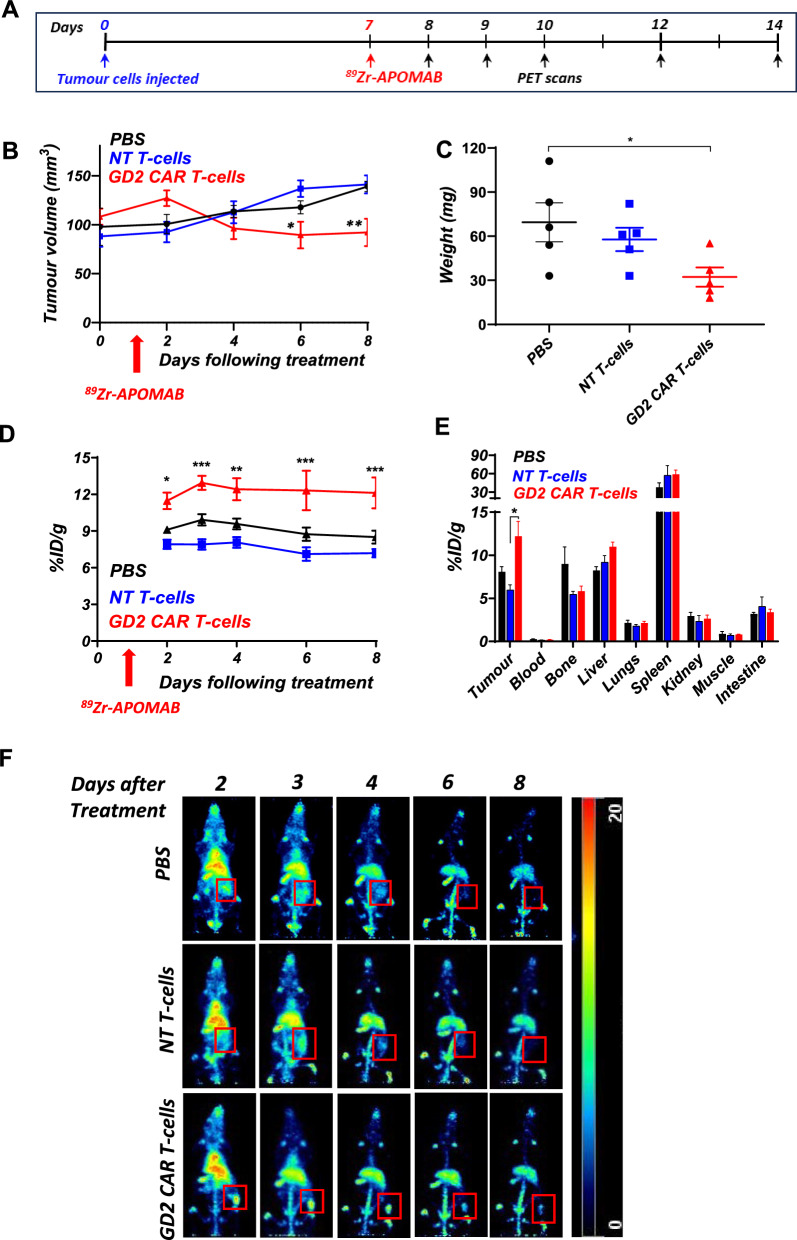
^89^Zr-APOMAB detects GD2-CAR-T cell mediated killing of LAN-1 tumours in vivo**.** LAN-1 tumours were established subcutaneously in NSG mice for 7 days before mice were treated with PBS, 10^7^ NT-T cells or GD2CAR-T cells intravenously. The following day mice were injected with ^89^Zr -labelled APOMAB and followed for tumour uptake for 8 days using PET imaging. **A** The timeline summarizes the treatment and imaging scheme of the study. **B** Tumour growth measured using callipers up to 8 days after treatment. **C** Tumour weights measured ex vivo. **D** Tumour uptake of ^89^Zr-APOMAB during the experiment was measured by live-animal PET imaging expressed as injected dose per g (%ID/g). **E** Biodistribution of the ^89^Zr-APOMAB at completion of the study (day 8 after treatment). **F** Representative Maximum Intensity Projections (MIPs) of whole-body PET images of a single mouse from each group are shown with red square showing tumour location and uptake (n = 5). All data points are means ± SEM, n = 5; *p* values determined by unpaired two-tailed t-test and 2-way ANOVA. * = *p* < 0.05, ** = *p* < 0.01, *** = *p* < 0.001 compared to control

Having demonstrated that APOMAB can detect immune-mediated cell death in vitro*,* we next wanted to see if we could use APOMAB-immunoPET to identify tumour responses to CAR-T cell therapy in vivo. LAN-1 subcutaneous tumours were established in immune-compromised NSG mice for 7 days before mice were given an intravenous injection with 10^7^ GD2-CAR-T cells, NT-T cells or PBS. CAR-T cell treatment significantly reduced tumour growth compared to NT-T cell treatment (*p* = 0.0053) and PBS (*p* = 0.0078), reducing the tumour size to 92.1mm^3^ ± 14.0 vs 141.6mm^3^ ± 9.3 in the NT-T cell treatment group and 139.1mm^3^ ± 5.2 in PBS control at day 8 after treatment (Fig. 2B) and was reflected in comparable and significant (*p* = 0.041) tumour weight reduction at the end of the study when compared to the PBS group (Fig. 2C). ^89^Zr-APOMAB was injected intravenously the day after CAR-T cell administration and PET imaging performed on days 2, 3, 4, 6 and 8 after treatment. Tumour uptake of ^89^Zr-APOMAB was significantly higher over the course of the experiment in mice treated with GD2-CAR-T cells compared to those treated with NT-T cells and PBS (Fig. 2D and 2E).

Interestingly, analysis of individual tumour-bearing mice illustrated in Supplementary Fig. 2 shows heterogeneity among the patterns of tumour growth after GD2-CAR-T cell therapy although all mice had evidence of some tumour accumulation of ^89^Zr-APOMAB after the treatment.

Analysis of biodistribution at the end of the study demonstrated significantly (*p* = 0.015) higher uptake of ^89^Zr-APOMAB in LAN-1 tumours after GD2-CAR-T cell treatment (12.2 ± 1.7% ID/g) compared to NT-T cell treatment (6.0 ± 0.6% ID/g) (Fig. 2E). These results show that tumour uptake of ^89^Zr-APOMAB corresponded to tumour shrinkage and suggested that APOMAB-immunoPET detected CAR-T cell-mediated death in vivo*.*

### APOMAB-immunoPET detects endogenous T-cell mediated tumour clearance

**Fig. 3:**
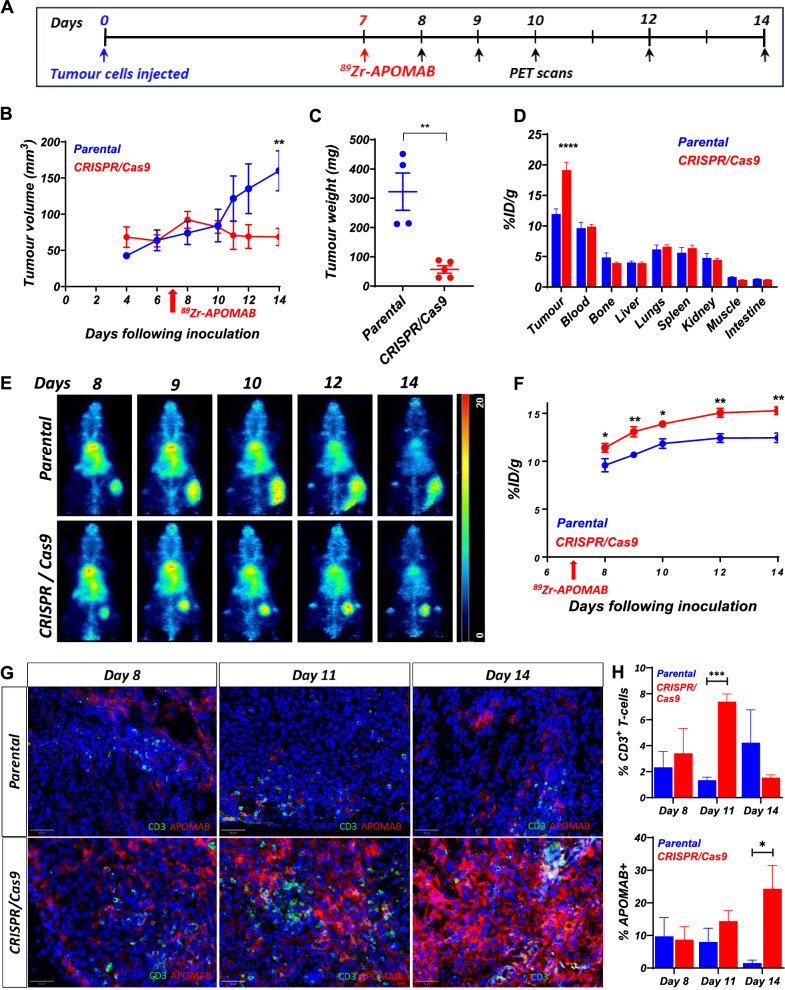
APOMAB detects endogenous T-cell mediated tumour clearance. BALB/c mice were injected subcutaneously with EMT6 parental cells or EMT6 cells expressing a CRISPR/Cas9 vector. Tumours were established for 7 days before mice were injected with ^89^Zr-APOMAB and analysed for tumour uptake for 7 days by PET imaging. **A** The timeline summarizes the treatment and imaging scheme of the study. **B** Tumour growth measured by callipers. **C** Tumour weights measured ex vivo. **D** Tissue biodistribution at the completion of the experiment measured by physical gamma counts. **E** Spatial MIPs of whole-body PET images of a representative mouse from each group. **F** Tumour uptake during the experiment measured using PMOD software. All data points are means ± SEM and *p* values were determined by two-way ANOVA ± SEM (n = 4–5). * = *p* < 0.05, ** = *p* < 0.01, **** = *p* < 0.0001. **G** Representative immunofluorescence images detecting CD3^+^ T-cells and APOMAB binding at selected time points after tumour inoculation. **H** Quantification of % of CD3^+^ T-cells and APOMAB^+^ cells in whole tumour section. Mean ± SEM, (n = 3), *p* values determined by unpaired two-tailed t-test. * = *p* < 0.05, *** = *p* < 0.001

As our GD2 CAR-T cell studies were performed in immunocompromised mice to prevent the rejection of engrafted human cells, we next wanted to determine if APOMAB-immunoPET could detect immune-mediated cell death in murine hosts with an intact immune system. To assess this, we used a syngeneic immunocompetent tumour model that had been developed in our laboratory [28]. We found that EMT6 murine mammary carcinoma tumours were spontaneously cleared by the immune system through expression of a lentiviral control CRISPR/Cas9 plasmid, which had integrated non-specifically in the EMT6 cell line and resulted in constitutive expression of the bacterially derived protein, Cas9. Others have shown that constitutive expression of Cas9 was immunogenic and led to tumour rejection and T-cell mediated clearance of the tumours in immunocompetent mice without the requirement of immune checkpoint blockade (ICB) or chemotherapy [29, 30]. EMT6 parental and EMT6 CRISPR/Cas9 subcutaneous tumours were established in BALB/c mice 7 days before administration of ^89^Zr-APOMAB. Mice were evaluated for ^89^Zr-APOMAB uptake by immunoPET for 7 days before tumours and organs were removed for biodistribution analysis. EMT6 parental and EMT6 CRISPR/Cas9 tumours demonstrated an equivalent level of growth for the first 8 days after inoculation, after which the EMT6 CRISPR/Cas9 tumours began to spontaneously shrink (Fig. 3B p = 0.006 and 3C *p* = 0.0025).

APOMAB-immunoPET imaging demonstrated significantly higher uptake of ^89^Zr-APOMAB in the EMT6 CRISPR/Cas9 tumours compared to the parental tumours (Fig. 3D) *p* < 0.0001 and 3F), with the greatest difference observed at the end of the study with tumour uptake of 15.1 ± 1.0% ID/g for the CRISPR/Cas9 group vs 11.4 ± 1.0%ID/g for the parental group. Biodistribution analysis demonstrated significantly higher binding of ^89^Zr-APOMAB in EMT6 CRISPR/Cas9 tumours (18.6 ± 3.1 ID/g) compared to the parental tumours (13.1 ± 1.6%ID/g) (Fig. 3D). To confirm T-cell infiltration, EMT6 parental and EMT6 CRISPR/Cas9 tumours were collected at 8-, 11- and 14-days post inoculation and CD3 expression in tumour sections was examined using immunofluorescence microscopy (Fig. 3G).

At day 11 a significant (*p* = 0.0007) increase in CD3 positive T-cells was observed in the CRISPR/Cas9 tumours compared to the parental tumours (7.4% ± 0.6% vs 1.3% ± 0.2%, Fig. 3H). To confirm APOMAB binding to dead cells within the tumour, mice were injected with APOMAB 24 h before tumour removal. There was a significant (*p* = 0.028) increase in APOMAB^+^ cells on day 14 in EMT6 CRISPR/Cas9 tumours compared to wild-type tumours (24.3% ± 7.1% for CRISPR/Cas9 vs 1.5% ± 0.9% parental, Fig. 3H). Collectively, these results demonstrate that APOMAB can detect cancer cell death in vivo after cytotoxic attack by endogenous T cells*.*

### APOMAB-immunoPET can be used to evaluate tumour responses to combination therapy using cytotoxic drugs and anti-PD-1 monoclonal antibody

**Fig. 4:**
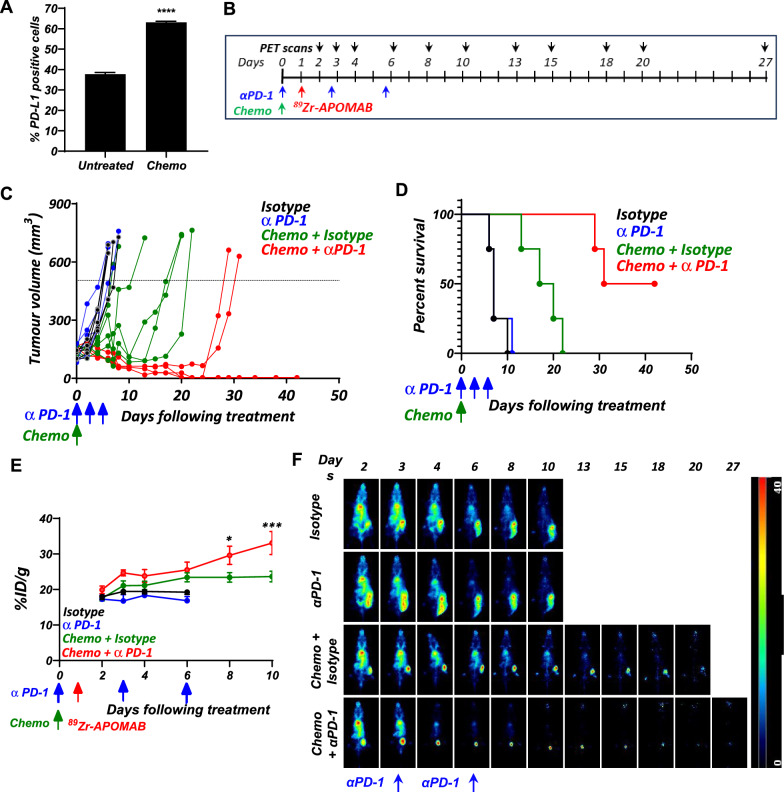
^89^Zr-APOMAB detects tumour response to combined chemotherapy and aPD-1 therapy in subcutaneous tumours of murine EL4 lymphoma**.** The EL4 murine lymphoma model was used to analyse APOMAB uptake following treatment with α-PD1 therapy. **A** EL4 cells were treated with etoposide and cyclophosphamide for 24 h and analysed by flow cytometry for PD-L1 expression. Data presented as percentage of PD-L1 positive cells, mean ± SEM. **B** The timeline summarizes the treatment and imaging scheme of the study. EL4 tumour-bearing C57BL/6 mice were administered with αPD-1 or its isotype control alone on days 0, 3 and 6 or in combination with chemotherapy alone on Day 0. **C** Tumour growth curves, **D** and survival of tumour-bearing mice. **E** Mice were injected with ^89^Zr-APOMAB on Day 1 and its uptake followed by PET imaging as quantified by PMOD software and, **F** demonstrated by MIPs of whole-body PET images of a representative mouse from each group. *P* values were determined by two-way ANOVA comparing the chemotherapy group with and without aPD-1. All data points are means ± SEM, n = 4/5. * = *p* ≤ 0.05, *** = *p* < 0.001

Developing tools that can provide an earlier indication of tumour responses to therapy is key to improving the clinical management of cancer. Having previously demonstrated that APOMAB can effectively detect tumour responses to chemotherapy [22], we wanted to test the ability of APOMAB-immunoPET to evaluate early tumour responses to combined chemotherapy and ICI. As murine EL4 lymphoma cells demonstrate an increase in surface PD-L1 expression following exposure to chemotherapy in vitro (Fig. 4B), a combination of chemotherapy and anti-PD-1 antibody was used and APOMAB uptake was measured by immunoPET. We have previously shown that EL4 tumours are highly chemo-responsive with post-apoptotic necrotic tumour cell death resulting in high levels of APOMAB uptake [17, 31]. Here, we also found that chemotherapy shrank EL4 tumours of treated mice compared to control untreated mice (Fig. 4C) and led to improved survival (Fig. 4D). Although anti-PD-1 alone had no obvious antitumour effect, the combination of chemotherapy with anti-PD-1 improved survival compared to chemotherapy alone (median survival 36.5 days vs 18.5 days) and 50% of mice (2/4) receiving the combination therapy were cured. The cured mice were rechallenged with EL4 cells 40 days after initial treatment. Although tumours established in naïve control mice, the rechallenged mice rejected the tumours, suggesting the formation of immunologic memory (data not shown).

APOMAB-immunoPET imaging analysis demonstrated that mice receiving a combination of chemotherapy and anti-PD-1 therapy showed significantly higher tumour uptake of ^89^Zr-APOMAB compared to chemotherapy alone, with highest tumour uptake observed on day 10 (33.1 ± 6.4%ID/g combination vs 23.6 ± 3.3%ID/g chemotherapy alone; *p* = 0.0005) (Fig. 4E). Uptake of ^89^Zr-APOMAB by the tumour was most obvious in the days following the final anti-PD-1 treatment (day 6) with %ID/g increasing from 25.5 ± 4.4 to 33.1 ± 6.5 on day 10, which corresponded to a reduction in tumour size (Fig. 4F).We hypothesize that this finding relates to residualization of ^89^Zr radiometal in tumour-associated macrophages that have engulfed ^89^Zr-APOMAB-bound dead tumour cells as described [23].

### ^89^Zr-APOMAB uptake in subcutaneous MC38 tumours after anti-PD-1 therapy

**Fig. 5:**
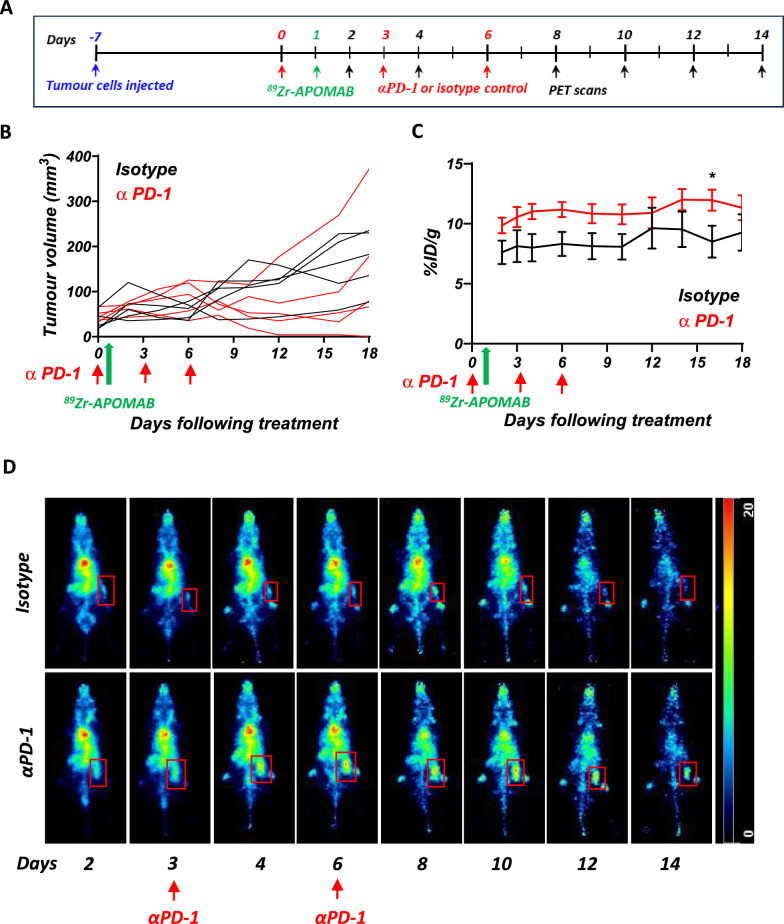
Increased uptake of ^89^Zr-APOMAB in MC38 tumours after aPD-1 blockade. C57BL/6 mice with established subcutaneous tumours of the MC38 cell line were treated with αPD-1 or isotype control antibodies on days 0-, 3- and 6, followed by injection with ^89^Zr-APOMAB. Tumour growth and ^89^Zr-APOMAB uptake was assessed over 18 days using PET imaging. **A** The timeline summarizes the treatment and imaging scheme of the study. **B** Tumour growth curves from MC38 tumour-bearing mice administered with αPD-1 or isotype control. **C** Tumour uptake of ^89^Zr-APOMAB in treated mice were measured using PMOD software. **D** Spatial MIPs of whole-body PET images of a representative mouse from each group are shown. Red box indicates location of tumour. All data points are mean ± SEM (n = 5) all days following treatment * = *p* ≤ 0.05

As EL4 tumour-bearing mice are unresponsive to anti-PD-1 alone, we examined the ability of APOMAB-immunoPET to detect tumour response to ICB using mice bearing syngeneic MC38 colon carcinoma, which has been shown to respond to anti-PD-1 therapy [32, 33]. MC38 subcutaneous tumours were established in C57/BL6 mice, and the mice were treated with anti-PD-1 therapy on days 0, 3 and 6. ^89^Zr-APOMAB was injected on day 1 and tumour uptake followed by PET imaging. Treatment of MC38 tumour bearing mice with anti-PD-1 antibody resulted in heterogeneous tumour responses, with delayed or reduced tumour growth, or both, in 4 out of the 5 mice (Fig. 5B) and shown for individual mice displayed in Supplementary Fig. 3. Here, the nonresponding mouse with the greatest tumour growth (Supplementary Fig. 3A) had lower tumour uptake of ^89^Zr-APOMAB when compared to the responding mice (Supplementary Fig. 3B). ^89^Zr-APOMAB uptake was consistently and significantly (*p* = 0.029) higher on day 15 among mice treated with anti-PD-1 compared to mice that were given the isotype control (Fig. 5C), with the highest uptake of ^89^Zr-APOMAB occurring 14 days after anti-PD-1 treatment (12.0 ± 2.0%ID/g with αPD-1 vs 9.5 ± 3.3%ID/g with isotype control). Representative whole body PET images show a persistent increase in tumour uptake of ^89^Zr-APOMAB in a mouse treated with anti-PD-1 compared to a mouse given isotype control antibody injections (Fig. 5D).

### APOMAB-immunoPET in a patient receiving combination immune checkpoint inhibitor therapy

**Fig. 6:**
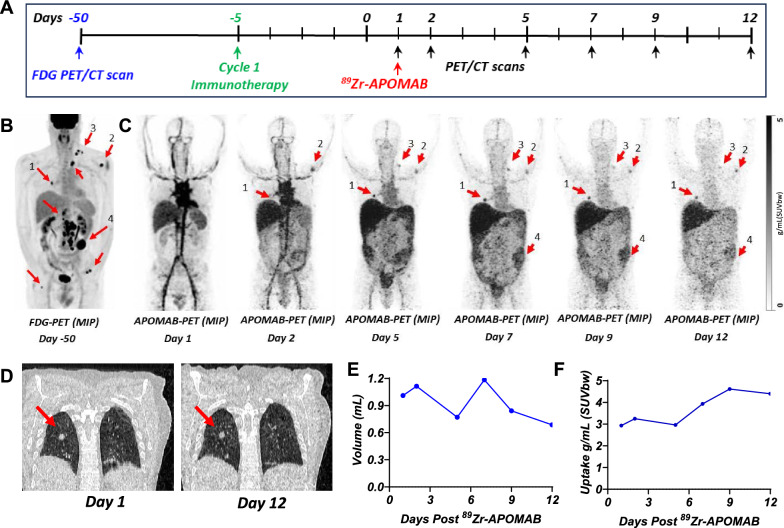
Variable post-immunotherapy tumour uptake of ^89^Zr-APOMAB and increased uptake demonstrated in a metastatic lung lesion. A metastatic melanoma patient received ipilimumab and nivolumab followed by an injection of 75MBq ^89^Zr-APOMAB five days later. PET/CT imaging was performed over the following 12 days. **A** The timeline summarizes the treatment and imaging scheme for the patient. **B** FDG-PET scan 50 days prior, with arrows identifying the metastatic lesions. **C** Spatial MIPs of APOMAB-PET images are shown. ^89^Zr-APOMAB positive lesions marked with red arrows. **D** Single slice coronal CT scans highlight the size and location of the pulmonary metastatic lesion at days 1 and 12. **E** Size of the metastatic lung lesion measured from the CT scans and quantified using PMOD. **F** Tumour uptake of ^89^Zr-APOMAB in the metastatic lung lesion measured using PMOD software following decay correction. Arrows locate the lesions in all images

Our previous preclinical investigations of APOMAB-immunoPET as an imaging method to diagnose tumour cell death and thus response to chemotherapy and radiotherapy led to initiation of a phase I clinical trial of ^89^Zr-APOMAB as a clinical imaging diagnostic. A trial patient, who had stage 4 V600E BRAF-mutant melanoma and received ipilimumab and nivolumab as ICI therapy. Five days later, the patient received an intravenous injection of ^89^Zr-APOMAB and PET/CT imaging was performed over the following 12 days to measure ^89^Zr-APOMAB uptake (Fig. 6A). A high-dose CT scan was done on day 5 to define an area of interest, which included a right-sided FDG-avid pulmonary lesion, for static PET scanning. Subsequently, the patient had cycles 2 and 3 of ipilimumab and nivolumab on days 14 and 35. On day 44, the patient developed symptomatic ascites requiring drainage and new peritoneal lesions thus denoting progressive disease. Subsequently, the patient was switched to BRAF/MEK inhibitor therapy that ultimately yielded a durable very good partial response now lasting two years.

Using a FDG-PET scan taken 50 days before immunotherapy as a reference to mark metastatic lesions (Fig. 6B), APOMAB-immunoPET demonstrated post-immunotherapy uptake of ^89^Zr-APOMAB in multiple lesions (Fig. 6C). Quantitative analysis of the right-sided FDG-avid pulmonary metastasis tumours revealed a reduction in tumour size that corresponded to an increase in ^89^Zr-APOMAB uptake 10 days post-immunotherapy (Fig. 6D–F). Other FDG-avid metastatic lesions had variable uptake of ^89^Zr-APOMAB over the 12 days of PET scanning. Although it is clear in this patient that early APOMAB-immunoPET did not predict a subsequent tumour response to immunotherapy, it is evident that the variable tumour uptake of ^89^Zr-APOMAB presaged an evolution of disease in which supradiaphragmatic lesions shrunk or remained stable while infradiaphragmatic lesions later progressed (data not shown).

## Discussion

In addition to the well characterized ability of APOMAB to detect chemotherapy-induced tumour cell death, we investigated its ability to detect immune-mediated cell death and identified ^89^Zr-APOMAB-immunoPET as a promising approach for early, non-invasive, and longitudinal detection of tumour cell death after T-cell based immunotherapies. Using four different preclinical tumour models, we detected increased tumour uptake of APOMAB by PET imaging after immune-mediated tumour cell death following CAR-T cell therapy, endogenous T-cell mediated tumour clearance, and ICB with and without chemotherapy. By detecting an endogenous biomarker of tumour cell death, this technique does not interfere with biological responses to therapy, nor does it require additional modifications to adoptive cell therapy products, thus broadening its application to the clinical investigation of immune-based anticancer therapies.

This study provides the first evidence that APOMAB can detect immune-mediated cell death, demonstrating a specific and dose-dependent induction of APOMAB binding following co-culture of GD2-CAR-T cells with GD2-expressing cancer cell lines. In vitro studies showed that APOMAB binding of dead cancer cells depended on antigen-specific induction of cytotoxicity mediated by GD2-CAR-T cells. Moreover, the extent of APOMAB binding of dead cancer cells in vitro corresponded to the extent of cytotoxicity mediated by GD2-CAR-T cells. This binding achieved levels approximating those shown for LAN-1 cell killing by apoptosis-inducing BH3 only mimetic drugs but lower than those caused by the DNA-damaging radiomimetic drug, cisplatin.

These results suggest how APOMAB binds dead cancer cells that have undergone T-cell mediated cell death. It is recognized that CAR-T cells most often cause cancer cell death by induction of apoptosis [34]. Hence, it is not unexpected that the level of APOMAB binding to dead cancer cells killed after CAR-T cell-mediated cytotoxicity is like that shown for the same target cells killed using pro-apoptotic BH3 only mimetic drugs. These findings are consistent with our earlier studies in which we found that the extent of APOMAB binding in vitro to dead cancer cells correlated with the extent of APOMAB co-localization with foci of the double strand DNA break (DSB) marker, γ-H2AX [23]. Similarly, we showed in Jurkat cells that activation of the extrinsic apoptotic pathway by Fas ligation resulted in increased APOMAB binding, which corresponded to detection of γ-H2AX and activated caspase-3 [19]. Here, we reason that, during apoptosis, γ-H2AX detection of DSB relates to activity of the Fas-induced Caspase-activated DNase complex [24], which contributes to DNA fragmentation and the formation of DSB and subsequently apoptotic bodies.

We hypothesise that the reduced APOMAB binding to cells dying after cytolytic attack compared to the higher binding observed in cells killed by the radiomimetic drug, cisplatin, results from the lower number and reduced persistence of DSB in the former compared to the latter. Furthermore, we do not believe that these APOMAB binding differences relate to differences in cell killing rates between cytotoxic chemotherapy and T-cell-mediated cytolysis because CAR-T-cell-mediated tumour cell death is described as prompt [34]. For example, using the MC38 tumour model, 4 to 5 days after administration of immune checkpoint blockade (ICB), mice demonstrated MRI-based imaging evidence of tumour cell necrosis, which was detected using a hyperpolarized ^13^C-fumarate probe, confirmed histologically, and associated with a CD8^+^ T-cell infiltrate [35]. And, in a recent clinical trial, rapid CAR-T cell killing in vivo of tumour cells in glioblastoma patients was inferred [36].

In vivo immunoPET studies showed that APOMAB bound GD2-expressing LAN-1 tumours after GD2-specific CAR-T cell-mediated cytotoxicity. Molecular imaging has been used by others to monitor CAR-T cell therapy including the use of reporter genes such as PSMA to track CAR T-cells [37], direct labelling of CAR T-cells [38] and detection of CAR-T cell activation [39]. By detecting an endogenous marker of tumour cell death rather than detecting CAR T-cells themselves, APOMAB-immunoPET may give an earlier, direct, and more realistic in vivo indication of tumour responses by measuring the extent of tumour cell death. Except for liver and spleen, which are well known to concentrate radiolabelled antibodies in NSG mice [20–22, 40], ^89^Zr-APOMAB only accumulated in post-CAR-T-treated tumour and not in other organs. In contrast to clearance of IV injected radiolabelled APOMAB over time in other organs, radiolabelled APOMAB accumulates with time in treated tumours for at least several reasons as demonstrated in previous studies: overexpression of the target antigen in cancer cells [41], and delayed clearance of dead tumour cells followed by tumour-associated macrophage-mediated uptake of radiolabelled APOMAB with subsequent residualization of the ^89^Zr label in macrophage lysosomes [15, 20, 42]. Finally, as this technique does not require modification to the CAR-T cell, it could be applicable to any CAR-T cell product, thus avoiding the time and investment associated with alterations to approved cellular therapies.

Then to mimic a clinical scenario more realistically, we used animals with fully functional immune systems for subsequent studies of APOMAB-mediated detection of immune-mediated tumour cell death. To do this, we employed a model developed in our laboratory, whereby forced expression of the bacterial derived Cas9 protein causes spontaneous tumour resolution in an immunocompetent mouse, a phenomenon also reported by others [29, 30]. This model provided the opportunity to evaluate the ability of APOMAB-immunoPET to detect T-cell mediated antitumour responses following an established time course in the absence of ICB or CAR-T cell therapy. ImmunoPET and biodistribution analyses demonstrated increased uptake of ^89^Zr-APOMAB by EMT6 CRISPR tumours that correlated with tumour shrinkage. We noted that peak of APOMAB binding to dead tumour cells (on day 14) occurred *after* the tumour T-cell infiltrate (on day 11), suggesting that APOMAB binds to dead tumour cells after endogenous T cell-mediated cytotoxicity had occurred. These findings are consistent with previous studies which used T-cell depletion to show that the rejection of Cas9-expressing tumours was T-cell mediated [29].

ICB has revolutionized immune-based therapy and is now offered as standard of care for a wide range of advanced cancers. Developing non-invasive and reliable methods for evaluating early tumour response remains an area of intense investigation. We assessed the utility of APOMAB-immunoPET for detecting tumour cell death after the combination of chemotherapy and ICB using the murine EL4 lymphoma model. Consistent with our previous studies [18, 21], ^89^Zr-APOMAB-immunoPET showed increased EL4 uptake after pro-apoptotic cyclophosphamide and etoposide chemotherapy [43]. Furthermore, tumour binding of APOMAB was significantly greater after the combination of chemotherapy and anti-PD-1 blockade than either treatment alone. Furthermore, combination therapy was associated with reduced tumour growth and increased survival of tumour-bearing mice. We found that in vitro exposure of EL4 cells to chemotherapy increased PD-L1 expression, thus potentially sensitizing tumour cells to PD-1/PD-L1 blockade, which is in keeping with the findings of another study showing that radiotherapy increased tumour PD-L1 expression and sensitized EL4 tumours to ICB [44].

In assessing the utility of APOMAB-immunoPET to detect immune-mediated tumour cell death after ICI therapy, we employed the MC38 tumour model in which sensitivity to PD-1 blockade has been shown [32] rather than the EL4 tumour model, which is not responsive to PD-1 blockade. We observed a reduction in tumour growth in 4 out of 5 mice treated with anti-PD-1 therapy, which corresponded to an increase in tumour uptake of ^89^Zr-APOMAB by PET imaging. In our hands, this model did not demonstrate a robust response to anti-PD-1 therapy as was reported in previous studies [32].

Varying responses to PD-1 blockade have previously been observed in the MC38 model and are, in part, explained by tumour-intrinsic resistance mechanism such as pro-tumour regulatory T cells and macrophages of the M2 phenotype [45, 46] as well as, for example, differences in the microbiome of the murine hosts under different agistment conditions [47]. Nonetheless, the observed rates of response in the MC38 model are not too dissimilar from those that may be observed among ICI-treated patients because of immune evasion [48]. Consequently, tumour uptake of APOMAB by immunoPET here observed as variable individual tumour responses in mice may reflect the interpatient heterogeneity of tumour shrinkage observed frequently in patients. Moreover, as a direct measure of the extent of immune-mediated tumour cell death, APOMAB-immunoPET may allow a more accurate assessment of immunotherapy-related tumour pseudoprogression than the conventional medical imaging modalities of CT, MRI, and FDG-PET/CT [8, 9].

To demonstrate the clinical relevance of our findings, we include data from a patient enrolled to our recently completed phase 1 clinical trial evaluating APOMAB-immunoPET in patients with advanced cancer (ACTRN12620000622909). In this patient, who had received combination ICI therapy as first-line treatment for stage 4 melanoma, ^89^Zr-APOMAB uptake was observed within a week in some of the FDG-avid metastatic lesions suggesting that immune-mediated tumour cell death may have occurred. Interestingly, in addition to the ^89^Zr-APOMAB-avid left-sided omental lesion, pooled radioactive fluid was observed in the patient’s pelvis by day 5 at the time of the high-dose CT scan, indicating the likelihood of dead melanoma cells detaching from the omental lesion into peritoneal fluid. Of note, the ^89^Zr-APOMAB-avid right-sided pulmonary metastatic focus reduced in size as ^89^Zr-APOMAB uptake increased over the 12-day scanning period. This finding indicates that this tumour shrank after immunotherapy thus increasingly concentrating the residualizing ^89^Zr radiometal, presumably engulfed within pre-existing or newly recruited tumour-associated macrophages in an expected and dynamic process of phagocytic clearance of dead cells [19, 20].

Uptake of ^89^Zr-APOMAB was not observed in all FDG-avid metastatic lesions. Given that this patient’s disease progressed after another six weeks, these early APOMAB-immunoPET data suggest not only was there an initial incomplete response to immunotherapy but also that inter-tumour responses to immunotherapy in a single patient can be heterogeneous.

In three cancer patients who were each given an intravenous injection of the CD8-specific radiolabelled minibody, ^89^Zr-crefmirlimab, 14–28 days after initiation of anti-PD1 therapy, post-treatment CD8-PET/CT scans detected increased tumour uptake of ^89^Zr-crefmirlimab by 24–48 h post-injection, which suggested tumour infiltration of CD8^+^ lymphocytes, and was later followed by tumour stabilisation or shrinkage [49].

In immunocompetent mice bearing syngeneic EMT6 tumours, a combination of ICI antibodies used to block PD-1 and stimulate ICOS resulted in the detection by the murine equivalent of ^89^Zr-crefmirlimab of a significant increase in intratumoral CD8 T cells 11 days after commencement of ICI therapy. In histological sections of tumours taken four days later (i.e., 15 days after commencement of ICI therapy), and compared to tumours taken from EMT6 tumour-bearing mice treated either with IgG control or ICOS agonistic antibody alone, the tumours were smallest with the highest degree of necrosis after the ICI antibody combination [50]. Interestingly, these data are consistent with what we had observed in our EMT6 Cas9 tumour model. We found peak tumour infiltration of endogenous T cells after 11 days, and peak tumour cell death, which was marked by previously intravenously administered naked APOMAB antibody, after 14 days.

Our data also show that evidence of immune-mediated tumour cell death can be detected using ^89^Zr-APOMAB-immunoPET many days after the T-cell mediated cytotoxicity, most likely because the long-lived radiolabel is retained in the phagolysosomes of professional scavenger cells, tumour-associated macrophages [23]. Moreover, mounting tumour accumulation of ^89^Zr-APOMAB appears to reflect the extent of immune-mediated tumour cell death in any one tumour. Current limitations of the APOMAB technique for tumour cell death detection in vivo include the need to match the approximate 3-day physical half-life of Zirconium-89 to the long circulating half-life of a fully intact APOMAB IgG1 monoclonal antibody. An equivalent small-molecule tracer labelled with a short-lived positron-emitting radioisotope, which could perform the same task of detecting immune-mediated tumour cell death, may allow imaging on the same day as the radiotracer injection and thus reduce the radiation hazard to patients and their caregivers. In addition, recruitment of additional patients receiving ICI or CAR-T cell therapies would strengthen our clinical findings by providing more data about APOMAB tumour targeting.

## Conclusion

Taken together, this study illustrates that APOMAB-immunoPET is a useful imaging modality for detecting tumour cell death mediated by the action of CAR-T cells, endogenous T cells, and T cells reinvigorated by immune checkpoint blockade.

To further evaluate its clinical utility, future clinical studies will aim to better characterize immune-mediated tumour cell death detected using APOMAB-immunoPET in more uniform study populations and in conjunction with other potential markers of responses to immune-based antitumour therapies.

## Supplementary Information

Additional file1 (PPTX 195 KB)

## Data Availability

The datasets used and/or analysed during the current study are available from the corresponding author on reasonable request.

## Abbreviations

7-AAD: 7-Aminoactinomycin D
ANOVA: Analysis of variance
ATCC: American Type Culture Collection
CAR: Chimeric antigen receptor
CD: Cluster of differentiation
CRISPR: Clustered regularly interspaced short palindromic repeats
CT: Computed tomography
DAPI: 4′,6-Diamidino-2-phenylindole
DMEM: Dulbecco’s Modified Eagle Medium
DMSO: Dimethyl sulfoxide
E:T: Effector:target
DSB: Double strand DNA breaks
FCS: Fetal calf serum
FDA: Food and drug administration
%ID/g: Percentage Injected dose per gram
IHC: Immunohistochemistry
i.p.i: intraperitoneal injection
ICB: Immune checkpoint blockade
ICI: Immune checkpoint inhibitors
IL: Interleukin
ICOS: Inducible T cell co-stimulator
irRECIST: Immune-related response evaluation criteria in solid tumours
La/SSB: Lupus-associated or Sjögren syndrome-B antigen
MFI: Median fluorescence intensity
MIP: Maximum intensity projection
MRI: Magnetic resonance imaging
NSG: NOD scid gamma-null
NT: Non-transduced
PBMC: Peripheral blood mononuclear cells
PBS: Phosphate buffered saline
PD-1: Programmed cell death protein 1
PD-L1: Programmed death-ligand 1
PET: Positron-emission tomography
iPERCIST: Immune PET response criteria in solid tumours
PI: Propidium iodide
RECIST: Response evaluation criteria in solid tumours
RPMI: Roswell Park Memorial Institute
SEM: Standard error of the mean
SUVbw: Standardized uptake value based on body weight
WT: Wild type
[^89^Zr]Zr^IV^: ^89^Zirconium
