# Supplementary material for: Detection of immune-mediated tumour cell death in vivo using Zirconium-89-labeled APOMAB®
Source: J Transl Med. 2025 Jun 12;23:651. doi: 10.1186/s12967-025-06684-z (PMC12164067; doi:10.1186/s12967-025-06684-z)
Supplement: Supplementary file 1 — Additional file1 (PPTX 195 KB) [file 12967_2025_6684_MOESM1_ESM.pptx]

## Slide 1
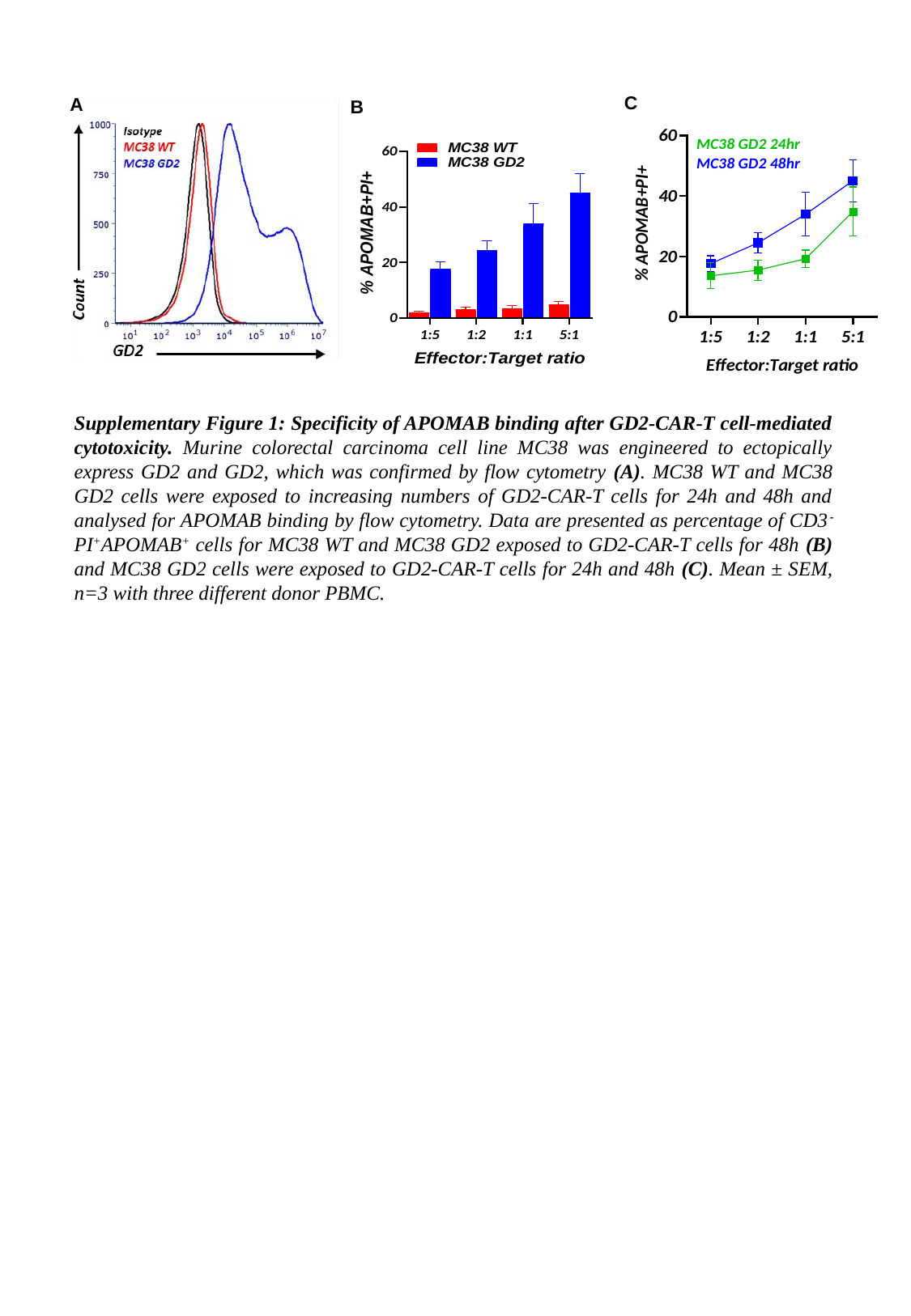

C
A
B
Supplementary Figure 1: Specificity of APOMAB binding after GD2-CAR-T cell-mediated cytotoxicity. Murine colorectal carcinoma cell line MC38 was engineered to ectopically express GD2 and GD2, which was confirmed by flow cytometry (A). MC38 WT and MC38 GD2 cells were exposed to increasing numbers of GD2-CAR-T cells for 24h and 48h and analysed for APOMAB binding by flow cytometry. Data are presented as percentage of CD3-PI+APOMAB+ cells for MC38 WT and MC38 GD2 exposed to GD2-CAR-T cells for 48h (B) and MC38 GD2 cells were exposed to GD2-CAR-T cells for 24h and 48h (C). Mean ± SEM, n=3 with three different donor PBMC.

## Slide 2
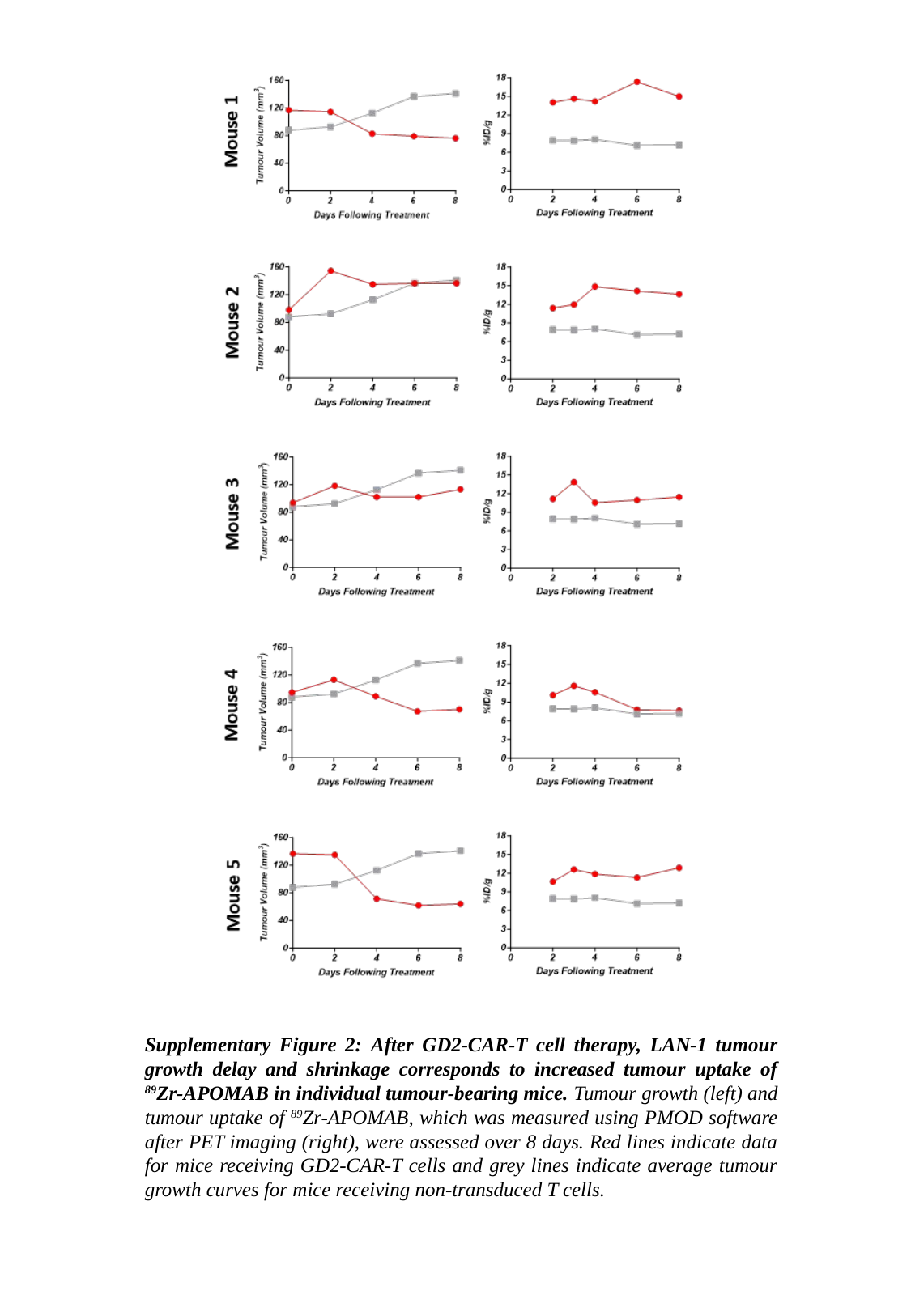

Supplementary Figure 2: After GD2-CAR-T cell therapy, LAN-1 tumour growth delay and shrinkage corresponds to increased tumour uptake of 89Zr-APOMAB in individual tumour-bearing mice. Tumour growth (left) and tumour uptake of 89Zr-APOMAB, which was measured using PMOD software after PET imaging (right), were assessed over 8 days. Red lines indicate data for mice receiving GD2-CAR-T cells and grey lines indicate average tumour growth curves for mice receiving non-transduced T cells.

## Slide 3
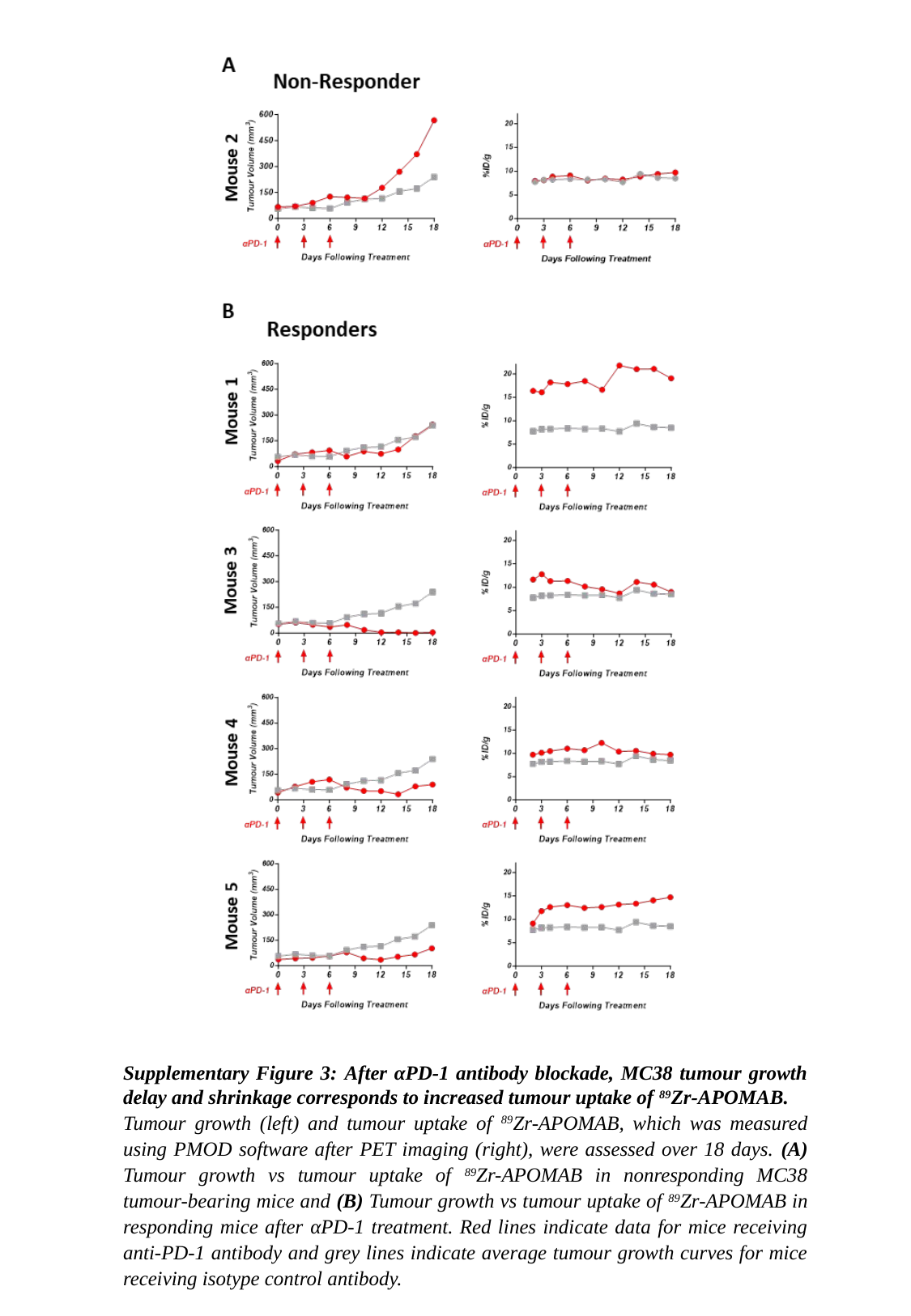

Supplementary Figure 3: After αPD-1 antibody blockade, MC38 tumour growth delay and shrinkage corresponds to increased tumour uptake of 89Zr-APOMAB.
Tumour growth (left) and tumour uptake of 89Zr-APOMAB, which was measured using PMOD software after PET imaging (right), were assessed over 18 days. (A) Tumour growth vs tumour uptake of 89Zr-APOMAB in nonresponding MC38 tumour-bearing mice and (B) Tumour growth vs tumour uptake of 89Zr-APOMAB in responding mice after αPD-1 treatment. Red lines indicate data for mice receiving anti-PD-1 antibody and grey lines indicate average tumour growth curves for mice receiving isotype control antibody.
